# Children’s neurodevelopment of reading is affected by China’s language input system in the information era

**DOI:** 10.1038/s41539-020-0062-0

**Published:** 2020-04-03

**Authors:** Wei Zhou, Veronica P. Y. Kwok, Mengmeng Su, Jin Luo, Li Hai Tan

**Affiliations:** 1 Beijing Key Laboratory of Learning and Cognition, School of Psychology, Capital Normal University, Beijing, 100037 China; 2 Center for Language and Brain, Shenzhen Institute of Neuroscience, Shenzhen, 518060 China; 3 College of Elementary Education, Capital Normal University, Beijing, 100037 China

**Keywords:** Neuroscience, Human behaviour

## Abstract

Communications through electronic devices require knowledge in typewriting, typically with the pinyin input method in China. Yet, the over utilization of the pronunciation-based pinyin input method may violate the traditional learning processes of written Chinese, which involves abundant visual orthographic analysis of characters and repeated writing. We used functional magnetic resonance imaging to examine the influence of pinyin typing on reading neurodevelopment of intermediate Chinese readers (age 9–11). We found that, relative to less frequent pinyin users, more frequent pinyin users showed an overall weaker pattern of cortical activations in the left middle frontal gyrus, left inferior frontal gyrus, and right fusiform gyrus in performing reading tasks. In addition, more frequent pinyin typists had relatively less gray matter volume in the left middle frontal region, a site known to be crucial for Chinese reading. This study demonstrates that Chinese children’s brain development in the information era is affected by the frequent use of the pinyin input method.

## Introduction

The rise of digital technology has penetrated every aspect of our lives, and it has especially changed the traditional mode of communication and learning. Instead of emphasizing the importance of handwriting skills, schools are promoting digital learning over the traditional pen-and-paper learning. Children are encouraged to read on electronic devices and are required to learn typewriting skills. Nevertheless, reading and writing skills are closely intertwined^1,2^. Literacy learning has become susceptible to frequent typewriting due to the popularization of electronic devices and learning platforms.

Learning to read involves the ability to recognize, understand, and use forms of written language, which is highly correlated with orthographic, phonological, and semantic skills. These linguistic skills provide a foundation for one’s reading ability^3–5^. While knowledge acquisition and electronic modes of communication have led to increased reliance on typewriting over handwriting in the digital age, it is critical to evaluate the effect of digital technology on reading development^6–11^. Unlike alphabetic writing systems that map graphic forms onto phonemes, logographic writing systems map graphic forms onto meanings. Chinese logographs are consisted of strokes and radicals packed into squared, visually complex spatial configurations^12,13^. Thus, this salient feature of the written characters demands intensive visuo-orthographic and visuospatial processing in Chinese reading acquisition^14–17^, and orthographic knowledge takes considerable effort and years of education to develop and master^18–20^. In Chinese, each character maps onto one syllable directly (e.g., 鱼 /yu2/; fish), and there is no single stroke or component that corresponds to the component phonemes of the syllable (e.g., either /y/, /u/, or the tone /2/). The traditional way of learning to read a character is by rote learning, which involves repeatedly copying the characters over and over. Through repetitive practice in character writing, children learn to deconstruct written forms into strokes and radicals, and regroup them into a whole linguistic unit. Therefore, successful Chinese reading acquisition requires proficient visuo-orthographic skills and formation of long-term motor memory of characters^20–22^. However, the most popular input method used in China—the pinyin method, is pronunciation-based. Pinyin is the romanization system of characters, which converts a pinyin sequence of whole characters to a list of Chinese characters sharing the same pinyin for typists to choose from. The pinyin input method is convenient and easy to learn, with almost no training. It allows users to input the pinyin of a single or multicharacter word and select the appropriate one from a list of characters sharing the same pinyin. Thus, the pinyin input method requires limited visuo-orthographic analysis of written Chinese characters. In Chinese primary schools, beginning readers learn and use pinyin to bridge the gap between spoken and written language, which complements the typical learning principle of Chinese reading and writing. However, overuse of pinyin for intermediate and adult readers may hinder reading development. A recent behavioral study in a large cohort of Chinese elementary school students (*n* = 5851) has revealed a negative correlation between children’s use of pinyin input and their reading scores, suggesting that increasing reliance on pinyin typing on e-devices could negatively affect children’s reading skill acquisition^23^.

Previous neuroimaging studies have observed that reading acquisition is capable of shaping both brain activation^24–27^ and brain anatomy^28–31^ of readers at different ages. However, little is known whether or not heavy reliance on typewriting over handwriting would interfere with children’s neural development associated with reading, especially in the brain regions involved in visuo-orthographic analysis of written words. Here, we used functional magnetic resonance imaging (fMRI) to examine the influence of the pinyin input method on children’s brain development associated with Chinese reading. Forty-five Chinese elementary school students participated in this study, which included 22 children who spent relatively more time (over 15 min per day) on pinyin typing on e-devices and 23 children who spent relatively less time (less than 15 min per day) (see Table 1). They performed four whole-brain fMRI runs, including reading comprehension of short stories, homophone judgment, orthographic judgment, and font-size judgment of Chinese characters. In the reading comprehension task, children read brief introductions of Hans Christian Andersen’s fairytale stories. The stories were presented sentence by sentence in each block and subjects were required to judge the correctness of two comprehension questions after each story. In the orthographic judgment task, subjects had to judge whether the two synchronously exposed characters had similar orthography (“材” and “林”) or not (“强” and “阵”). In the homophone judgment task, subjects decided whether the presented character pairs were homophones (“师” and “诗” both pronounced shi1) or not (“球” and “核” pronounced qiu2 and he2). In the font-size judgment task, subjects were asked to judge whether the presented character pairs had the same physical size. Subjects made button responses with their right hand in all four runs. All mentioned tasks had the same control scan, in which subjects were instructed to fixate on the fixation crosshair presented at the center of the screen, and no feedback was required. In addition, children’s structural 3D MRI images were analyzed to determine possible brain structure differences between groups. The final number of children included in each analysis (see “Methods” for criteria of subject inclusion) were reported in Table 1.

**Table 1:** Subjects’ demographic information and reading scores.

	Usage of pinyin typing		*P*-value
	More	Less	
No. of children	22	23	—
Age (month)	124	123	0.700
Sex (boy/girl)	9/13	14/9	0.181
Left/right-handed	0/22	0/23	—
Parental education (maximum score is 10)	6.0	6.2	0.627
Average school grades	4.6	4.4	0.409
Nonverbal WISC subtest: blocked design (scaled score)	10	10	0.731
Character recognition (maximum score is 150)	105	116	0.031
Single-character word-reading fluency (items per minute; *n* = 34)	105	119	0.048
Two-character word-reading fluency (items per minute; *n* = 34)	79	98	0.003
Single-pinyin item reading fluency (items per minute; *n* = 34)	46	54	0.213
Two-pinyin item reading fluency (items per minute; *n* = 34)	21	24	0.306
Orthographic awareness (items per minute)	40	43	0.202
Phonological deletion (maximum score is 28)	16	17	0.442
Daily pinyin typing time (minute)	65	9	<0.001
Daily total time on devices (minute)	117	26	<0.001
Daily writing time of Chinese characters (minute)	86	87	0.896
Daily offline book reading time (minute)	61	66	0.687
Number of subjects for the analysis of reading comprehension after exclusion of invalid data	21	21	—
Number of subjects for the analysis of the homophone, orthographic, and font-size judgment tasks after exclusion of invalid data	20	21	—
Number of subjects for the analysis of VBM after exclusion of invalid data	20	19	—

Our subjects’ reading skills were assessed through a battery of behavioral tasks, including Chinese character recognition, orthographic awareness, and phoneme deletion^22,32^. All children completed a questionnaire regarding their total time spent on e-devices per day, character writing time per day, offline book reading time per day (see “Methods” for details) and their parents’ education levels (i.e., below primary level, grade 1 to grade 3 in primary school, grade 4 to 6 in primary school, middle school level, technical secondary school level, high school level, associate’s level, bachelor’s level, master’s level, and doctorate level). In order to make direct comparisons between children’s performance in reading pinyin and characters, we managed to call back 34 (17 more frequent vs. 17 less frequent typists) out of 45 children to complete the single-character word reading (160 items), two-character word reading (180 items), single-pinyin item reading (160 items), and two-pinyin item reading (180 items) tasks (see “Methods” for details). These tasks served as an objective measure of children’s reading fluency.

## Results

### Behavioral data

As shown in Table 1, there were significant group differences in character recognition (*t*(43) = −2.23, *p* = 0.031), total time spent on e-devices (*t*(43) = 4.96, *p* < 0.001), and daily pinyin typing time (*t*(43) = 6.36, *p* < 0.001). These two groups were matched in age, sex, handedness, parental education (the average of father and mother education levels), average school grades, character writing time per day, and offline reading time per day (*ps* > 0.05).

The reading fluency test of characters and pinyin showed that more frequent pinyin users produced lower scores in both single-character (*t*(32) = −2.06, *p* = 0.048) and two-character word reading (*t*(32) = −3.22, *p* = 0.003) than less frequent pinyin users, but there were no significant group effects in single-pinyin and two-pinyin item reading (*ps* > 0.05).

**Fig. 1:**
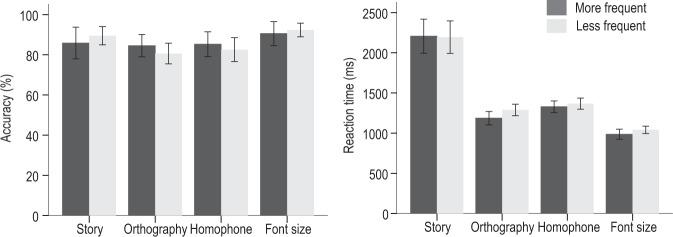
The behavioral performance of more frequent and less frequent typists in each fMRI task. The left panel shows the mean accuracy rate and the right shows the mean reaction time. Erro bars depict SEM.

Behavioral data from the in-scanner tasks (see Fig. 1) showed that there were no significant group differences in accuracy and reaction time in reading comprehension (accuracy: *t*(40) = −0.83, *p* = 0.412; reaction time: *t*(40) = 0.079, *p* = 0.937), orthographic judgment (accuracy: *t*(39) = 1.08, *p* = 0.285; reaction time: *t*(39) = −1.97, *p* = 0.056), homophone judgment (accuracy: *t*(39) = 0.66, *p* = 0.516; reaction time: *t*(39) = −0.79, *p* = 0.433), and font-size judgment (accuracy: *t*(39) = −0.55, *p* = 0.585; reaction time: *t*(39) = −1.45, *p* = 0.155) tasks.

### Imaging data

#### Reading comprehension task

**Fig. 2:**
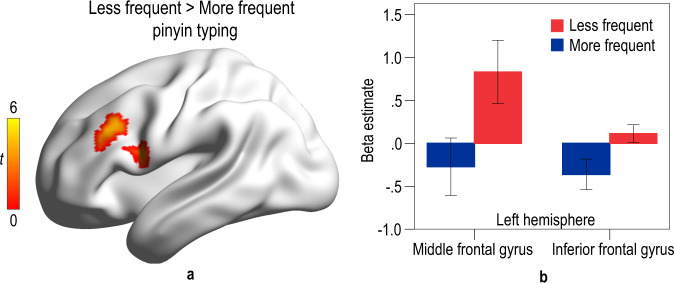
The effect of pinyin typing on brain activation in reading comprehension. **a** Brain regions showing significant group effect in reading comprehension (permutation test with TFCE correction at *p* < 0.05, cluster size >20). The color bar denotes *t*-value. **b** The brain activations (± 1.96SE) of the left middle frontal gyrus and left inferior frontal gyrus in each group.

Less frequent pinyin users showed stronger activations in the left middle frontal gyrus (BA 9/46; *x* = −48, *y* = 27, *z* = 30; cluster size = 69) and left inferior frontal gyrus (BA 44/45; *x* = −42, *y* = 15, *z* = 12; cluster size = 46) after a voxelwise and nonparametric permutation test with Threshold-Free Cluster Enhancement (TFCE) correction (*p* < 0.05, cluster size > 20) (see Fig. 2a and Table 2; please refer to Supplementary Fig. 3 for activation within each group)^33–35^. More frequent pinyin typists did not show stronger activation in any brain region relative to the less frequent pinyin typists. We extracted the parametric estimates (beta values) from spheres (radius = 6 mm) centered on the peak coordinate in the left middle frontal gyrus and left inferior frontal gyrus for each group (see Fig. 2b). In addition, we found significant and positive correlations between the character recognition score and the brain activations of the left middle frontal gyrus (*r*(42) = 0.477, *p* = 0.001) and left inferior frontal gyrus (*r*(42) = 0.405, *p* = 0.008) (see Supplementary Fig. 5).

**Table 2:** Coordinates of activation peaks in four fMRI tasks.

Regions	MNI coordinates			*T*-value
	*x*	*y*	*z*	
Reading comprehension > rest				
*Typing more*				
Left lingual gyrus	−15	−93	−12	14.94
Left temporal pole	−48	15	−21	8.23
Left middle temporal gyrus	−57	−24	−3	6.31
Right lingual gyrus	18	−87	−9	18.72
*Typing less*				
Left lingual gyrus	−15	−90	−15	14.46
Left middle temporal gyrus	−54	−30	0	8.88
Left superior temporal gyrus	−57	0	−12	8.19
Left fusiform gyrus	−42	−45	−24	7.19
Left inferior frontal gyrus	−51	24	18	6.93
Left middle frontal gyrus	−39	3	54	6.42
Left supplementary motor area	−6	12	63	6.37
Left pallidum	−21	6	0	6.25
Left medial frontal gyrus	−6	60	33	5.22
Right lingual gyrus	18	−90	−15	11.02
Right temporal pole	48	18	−18	5.56
*Typing less > typing more*				
Left inferior frontal gyrus	−42	15	12	5.12
Left middle frontal gyrus	−48	27	30	4.48
Orthographic judgment > rest				
*Typing more*				
Left lingual gyrus	−21	−96	−15	9.45
Right inferior occipital cortex	30	−90	−3	12.45
*Typing less*				
Left inferior occipital cortex	−27	−90	−9	11.63
Left cerebellum	−48	−63	−21	8.39
Left middle frontal gyrus	−42	9	30	6.52
Left superior parietal lobe	−24	−66	45	6.20
Right fusiform gyrus	42	−69	−18	9.04
*Typing less > typing more*				
Right fusiform gyrus	36	−39	−18	4.70
Right fusiform gyrus	27	−36	−21	4.42
Homophone judgment > rest				
*Typing more*				
Left lingual gyrus	−21	−96	−15	9.18
Right inferior occipital cortex	39	−78	−12	10.39
*Typing less*				
Left inferior occipital cortex	−27	−90	−6	9.70
Left inferior temporal gyrus	−42	−36	−12	5.37
Right inferior occipital cortex	36	−84	−15	6.89
Right cerebellum	3	−60	−21	5.30
Font-size judgment > rest				
*Typing more*				
Left middle occipital cortex	−27	−96	0	10.36
Left fusiform gyrus	−45	−51	−18	6.26
Right inferior occipital cortex	30	−90	−6	13.53
*Typing less*				
Left inferior occipital cortex	−27	−90	−6	7.77
Right inferior occipital cortex	30	−84	−6	8.20

Note. Coordinates are referred to the peak *t*-values for each region (permutation test with TFCE correction at *p* < 0.05).

**Fig. 3:**
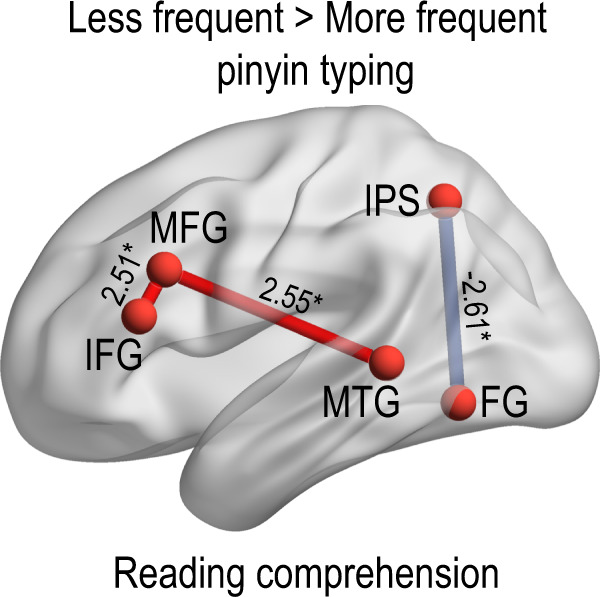
The effect of pinyin typing on functional connectivity. The *t*-values for group comparison of functional connectivity strength are presented next to the connection line. The red line indicates a positive contrast value. The blue line indicates a negative contrast value. *q_corrected_ < 0.05 (FDR-corrected for multiple comparisons). FG fusiform gyrus, IFG inferior frontal gyrus, IPS intraparietal sulcus, MFG middle frontal gyrus, MTG middle temporal gyrus.

To examine whether pinyin typing interfered communication among brain regions within children’s reading network, we conducted region-of-interest-based functional connectivity analyses. Regions of interest were spheres (radius = 6 mm) centered on the peak MNI coordinates of reading-related regions found in previous studies (listed in Table 3)^36–38^. In the reading comprehension task, less frequent pinyin users had stronger functional connections between the left middle frontal gyrus and left inferior frontal gyrus and between the left middle frontal gyrus and left middle temporal gyrus, and a weaker functional connection between the left intraparietal sulcus and left fusiform gyrus (*qs*_*corrected*_ < 0.05) as compared with more frequent pinyin users (see Fig. 3).

**Table 3:** The seed regions defined in the functional connectivity analyses.

Brain region	MNI coordinates		
	*x*	*y*	*z*
Left middle frontal gyrus	−49	20	29
Left inferior frontal gyrus	−53	27	16
Left superior temporal gyrus	−53	−31	9
Left middle temporal gyrus	−59	−42	3
Left intraparietal sulcus	−30	−58	48
Left fusiform gyrus	−45	−62	−8
Right fusiform gyrus	45	−62	−8

### Orthographic judgment task

**Fig. 4:**
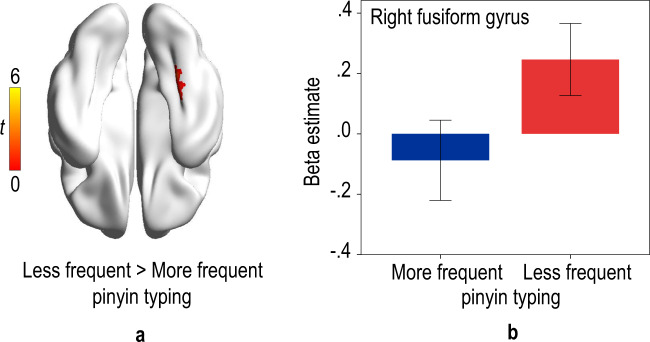
The effect of pinyin typing on brain activation in orthographic judgment. **a** Brain region showing a group effect in orthographic judgment (permutation test with TFCE correction at *p* < 0.05). The color bar denotes *t*-value. **b** The brain activation (± 1.96SE) of the right fusiform gyrus in each group.

There was a significant group effect on brain activation in orthographic judgment. Children who spent less time on pinyin typing produced greater activation in the right fusiform gyrus (BA 37; *x* = 36, *y* = −39, *z* = −18, cluster size = 10 and *x* = 27, *y* = −36, *z* = −21, cluster size = 8) after permutation test with TFCE correction (*p* < 0.05) in orthographic judgment (Fig. 4a). The mean parametric estimate from the 6-mm sphere centered on the peak coordinate identified in the right fusiform gyrus (*x* = 36, *y* = −39, *z* = −18) for each group is shown in Fig. 4b. In addition, there was a positive correlation between the score of orthographic awareness and the activation of the right fusiform gyrus (*r*(41) = 0.367, *p* = 0.018) (see Supplementary Fig. 6). There was no significant group difference in the strength of functional connections in the orthographic task (*qs*_corrected_ > 0.05).

### Homophone judgment task and font-size judgment task

The group effects on brain activation and functional connectivity in both tasks were not significant.

### Structural brain difference

**Fig. 5:**
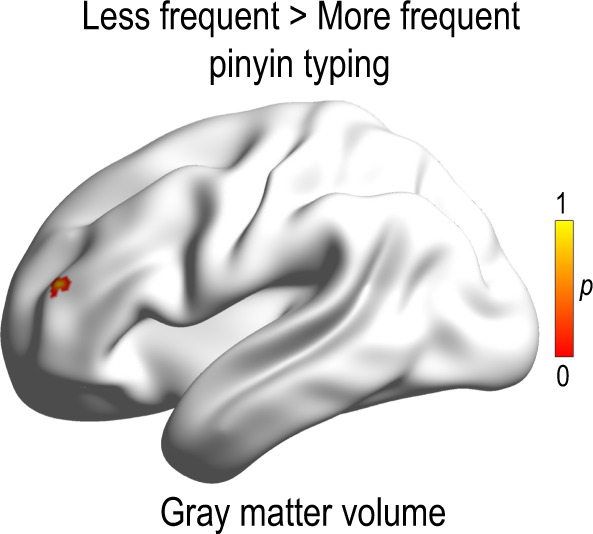
Structural difference between less frequent and more frequent pinyin typists in the language-related cortical region. The brain region showing significant group effect in VBM analysis (permutation test with TFCE correction at *p* < 0.05). The color bar denotes *p*-value.

We performed a voxel-based morphometry (VBM) analysis to further investigate whether there were structural differences between the two groups of children in the brain regions responsible for Chinese reading. Since previous studies have indicated the importance of the left middle frontal gyrus in Chinese reading and reading acquisition, we examined the group difference in this brain region following a priori hypothesis as in a past study^30^. For the statistical analysis, a small volume correction analysis procedure was applied to the left middle frontal gyrus (BA 9, *x* = −32, *y* = 31, *z* = 28, Talairach atlas; 20 mm region of interest), which was identified in reading in Chinese children^30^. We found that children who spent less time using the pinyin input method had significantly higher gray matter volume in the left middle frontal gyrus (BA10; MNI coordinate: *x* = −36, *y* = 46, *z* = 18; cluster size = 13) relative to those who spent more time on pinyin typing after permutation test with TFCE correction at *p* < 0.05 (see Fig. 5).

### Further analysis of pinyin typing effects

We have conducted a regression analysis with sex, age, grade, nonverbal IQ, parental education, head motion (i.e., the average of six rigid-body motion parameters parameters), offline reading time, total time on e-devices, character recognition score, and pinyin typing group as covariates. After regressing out the effect of these possible confounding variables, we found that the following effects of pinyin typing on neurodevelopment remained significant: brain activations in the left middle frontal gyrus (*β* = 0.482, *p* = 0.001), left inferior frontal gyrus (*β* = 0.589, *p* < 0.001), and right fusiform gyrus (*β* = 0.529, *p* < 0.001), gray matter volume of the left middle frontal gyrus (*β* = 0.492, *p* = 0.001), and functional connectivity strengths of left middle frontal gyrus–left inferior frontal gyrus (*β* = 0.590, *p* = 0.001), left middle frontal gyrus–left middle temporal gyrus (*β* = 0.374, *p* = 0.015), and left intraparietal sulcus–left fusiform gyrus (*β* = −0.382, *p* = 0.013).

## Discussion

This study has combined several approaches including fMRI, MRI, and brain-behavior correlations to examine whether the utilization of the pronunciation-based pinyin input method affected Chinese children’s reading development. Our findings have demonstrated that frequent usage of pinyin typing and digital devices were associated with overall weaker cortical activations, weaker functional connections, and less gray matter volume within the Chinese reading cortical network. In general, pinyin typing may have a negative impact on intermediate readers’ reading acquisition.

Previous neuroimaging studies have consistently indicated the critical role of the left prefrontal cortex in reading^39–41^. The left inferior frontal gyrus is involved in semantic processes at the word^27,42–47^ and sentence level^48–50^, and the left middle frontal gyrus is particularly important in Chinese reading and reading acquisition^16,30,51–54^. In this study, the left middle frontal gyrus is likely engaged in visuospatial computation, orthography-to-phonology transformation, and orthography-to-semantics mapping. Chinese children with dyslexia have shown reduced brain activity and gray matter volume in the left middle frontal gyrus^16,30,54^. This region is also known to be crucial in the coordination of cognitive resources in working memory to establish reading–writing connection^2,21^. Chinese learners who have practiced handwriting exhibited stronger activations in the left middle frontal gyrus in comparison with those who did not^21^. Fluent reading requires an integrated reading circuit that links orthography, pronunciation, and meaning. However, pinyin typing relies heavily on the phonological encoding without sufficient analysis of the internal orthographic structures of written characters. The linguistic mappings of orthography to phonology and orthography to meaning developed through handwriting could be weakened by the increasing reliance of the phonological strategy used in pinyin typing.

Meanwhile, the weaker activation in the right fusiform gyrus observed in more frequent pinyin typists suggests that pinyin typing may interfere with the learning of visuospatial properties of characters. The right visual cortex has a role in elaborated visuospatial analysis of various strokes within the logographic character. Previous research has illustrated that the right visual system was greatly involved in Chinese reading, but not in English^53^. Skilled adult readers also showed stronger activation in the right occipital cortex than children in Chinese word reading^55^. When children get used to the pinyin input method, they start to rely on pronunciations instead of strokes and radicals to generate characters. Thus, the association between character forms and pronunciation of the characters deteriorates and weakens the cortical activation in the visual region. Chinese college students who relied on the orthographic-based input method over the pronunciation-based input method showed better performance in Chinese reading tasks^9^, thus suggesting that successful Chinese reading and writing are strongly correlated with proficient knowledge of visual word form of characters. The positive correlation between orthographic awareness and the activation in the right fusiform gyrus has corroborated this assumption. We currently did not find a reliable effect of typing experience on the brain activation in the homophone judgment task. As there are a large number of characters being homophones in the Chinese writing system, both handwriting and typing in pinyin require phonological processing. The question of whether and how typing experience influences brain activation in a phonological task merits further investigation.

In language development, we learn how to speak before learning to read. Thus, the pinyin system serves as an important tool in bridging the gap between vocabularies learnt through the spoken language with the written scripts, and it plays an essential role in early reading development. Beginning readers (6–8 years old) rely on pinyin to aid reading in Chinese primary schools, as revealed in past studies^56,57^. After several years of character reading and writing practices, intermediate readers (9–11 years old) start to develop the concepts of radicals and character components^19,20^. Since then, their dependence on pinyin is expected to drop because pinyin cannot replace Chinese characters in any way^58^. The over-reliance on pinyin for intermediate and skilled Chinese readers could slow down the learner’s path to build a solid foundation for character recognition and reading fluency.

Together, our findings have demonstrated that the pinyin input method is associated with functional and structural changes to intermediate Chinese readers’ brains, namely, in the core regions for Chinese reading, including the left inferior and middle frontal gyri, and the right fusiform gyrus. We have also shown that the frequent usage of the pinyin input method led to a weaker dynamic connection among reading regions in these children. Our results suggest that the popular pinyin input method is related to Chinese children’s poor reading development. Chinese children are facing literacy challenges in the digital age. To preserve visuo-orthographic encoding of Chinese characters, we would recommend to restrict the use of pinyin input, and promote orthography-based input methods including handwriting.

## Methods

### Subjects

Forty-five right-handed, native Mandarin speaking children (mean age 10.2 years, ±1.0 years; 22 females) participated in this study, who were fourth to sixth graders in primary school. They were strongly right-handed as measured by the Edinburgh Handedness Inventory^59^. All children had normal or corrected-to-normal vision, and none had any history of neurological or psychiatric illness. This study was approved by the Research Ethics Committee at the Capital Normal University. Each child and parent signed an informed written consent before the experiment. Subjects were divided into two groups (more frequent pinyin typing group, *n* = 22; less frequent pinyin typing group, *n* = 23) according to their daily pinyin typing time (more or less than 15 min) on the digital devices each day (see “Questionnaire on digital device usage” for details). Three children were excluded from the reading comprehension task analysis (one more frequent and two less frequent pinyin typists), and three children were excluded from the analyses of orthographic judgment, homophone judgment, and font size judgment tasks (two more frequent and one less frequent typists) due to severe head movement (one of six rigid-body motion parameters exceeded 3 mm). One more child in less frequent typing group was excluded from the analyses of orthographic judgment, homophone judgment, and font-size judgment tasks due to over 30% missing behavioral responses. Six children (two more frequent and four less frequent pinyin users) were excluded from the analysis due to severe head movement during the structural scan. There was no significant group difference in head motion (i.e., the average of six rigid-body motion parameters) in all fMRI tasks (*ps* > 0.05). All children completed a questionnaire on digital device usage and a battery of reading achievement tasks, including Chinese character recognition, orthographic awareness, and phonological deletion. These behavioral tasks were completed prior to the fMRI experiment. In addition, we managed to call back 34 (17 more frequent vs. 17 less frequent typists) out of 45 children to complete the reading fluency tests of characters and pinyin.

### Questionnaire on digital device usage

All children completed a questionnaire regarding daily usage of digital devices under the supervision of their parents. The parents did not decide or pick the answer for their kids, instead, their presence was only to make sure that their children understood all questions listed on the questionnaire and to confirm the validity or relevance of their answers. These questions were adapted from Tan et al.^23^, which included (i) the number of digital devices that children had (i.e., computer, tablet, and cell phone) at home; (ii) the average time they spent each day using digital devices; (iii) the average time they spent each day typing pinyin on digital devices; (iv) the average time they spent each day on writing Chinese characters (e.g., doing homework or writing letters) with a pen at home; (v) the average time they spent each day on offline reading (e.g., reading textbooks or extracurricular books) at home. The time was assessed according to a 6-point scale: 0 min, 15 min, 30 min, 60 min, 90 min, and 120 min. The aggregated time on pinyin typing and using digital devices was the sum of selected options for typing pinyin using computer, tablet, and cell phone.

### Behavioral tasks

*Nonverbal IQ test*: Nonverbal IQ was estimated based the nonverbal Wechsler Intelligence Scale for Children-IV (WISC-IV) subtest: Block Design (*Mean* = 10, *SD* = 3)^60–62^. Scaled IQ scores were calculated based on age-specific norms.

*Character recognition*: Children were asked to name a list of 150 single characters, among which 140 items were selected from Chinese language textbooks in primary school, and another 10 items that did not appear on textbooks^22,32^.

*Orthographic awareness*: Children were shown a list consisted of 70 items, among which 10 items were black-and-white line drawings that contained no conventional stroke patterns, 10 items were compound noncharacters with spatially transposed components, 10 items were compound noncharacters with wrong components, 10 items were pseudocharacters that consisted of real components of Chinese with components in their legal positions and 30 real Chinese characters^22,32^. Children were asked to judge whether each item was a real character or not as quickly as possible.

*Phoneme deletion*: Children were instructed to produce a new syllable by taking away the target phoneme from a monosyllabic Chinese word^22,32^. For example, given the syllable *bei4*, children were asked to delete the *b* sound. The answer, in this case, is *ei4*. Children completed 28 trials which required the deletion of the initial, middle, or last phonemes.

*Reading fluency*: The measures of reading fluency included timed single-character word reading, two-character word reading, single-pinyin item reading, and two-pinyin item reading. The character and pinyin reading tasks measured the total number of characters and pinyin items that the children could read as accurately and fast as possible within 1 min. For the single-character word-reading test, we constructed a list of stimuli with 160 characters which were used in the orthographic and homophone judgment tasks in the current fMRI experiment. For the two-character word-reading test, we adopted the list of word stimuli used in a previous children study^32^ that contained 180 two-character words in total. The stimuli used in the single-pinyin and two-pinyin item lists were pinyin transcriptions of the single-character and two-character word lists, respectively. The presentation of pinyin transcriptions was randomized to ensure that the children did not notice the items in the character- and pinyin-reading tasks were familiar.

### fMRI procedures and reading tasks

Each child completed four fMRI task runs, including reading comprehension, orthographic judgment, homophone judgment, and character font-size judgment. In the reading comprehension task, children were required to read the story silently and judge the correctness of two comprehension questions by pressing the button with their right hand after each story. The baseline condition was rest, in which fixation crosshairs were displayed in the center of the screen, and no feedback response was required. Four brief introductions of Hans Christian Andersen’s fairytale stories in the Chinese extracurricular book^63^ were edited as the materials for the reading comprehension task. Each story had ten sentences with an average of 13.6 (SD = 1.5) characters for each sentence (see Supplementary Fig. 1). All characters in the stories have been listed as learning materials in textbook from Grades 1 to 4. There were four blocks in the reading comprehension task. One story was presented sentence by sentence in each block. According to a pilot measurement of children’s reading speed, each sentence was presented for 3500 ms and followed by a blank screen for 500 ms. Each question was presented for 5000 ms and followed by a blank screen for 1000 ms. The task started with 14-s of rest, and each reading comprehension block was alternated with 14-s rest blocks. Subjects practiced to read two stories and answered the questions before scanning. The stories used in the practice session did not appear in the actual experiment.

In the orthographic judgment task, subjects were asked to decide whether the presented character pairs had similar orthography (“材” and “林”) or not (“强” and “阵”). The baseline condition was rest, in which fixation crosshairs were displayed in the center of the screen. Subjects responded by pressing a button with their right hand. Forty pairs of characters were selected from the primary school textbooks as stimuli. Half of the character pairs had similar orthography, while the other half did not.

In the homophone judgment task, subjects judged whether the visually presented character pairs were homophones (“师” and “诗” both pronounced shi1) or not (“球” and “核” pronounced qiu2 and he2). The baseline condition was rest, in which fixation crosshairs were displayed in the center of the screen. Subjects responded by pressing a button with their right hand. Forty pairs of characters were selected from the primary school textbooks as stimuli. Half of the word pairs were homophone, and the other half were not.

In the font-size judgment task, subjects were required to decide whether the presented word pairs had the same physical size. Subjects responded by pressing a button with their right hand. Forty pairs of characters were selected from the primary school textbooks as stimuli. Half of the character pairs had the same physical size, and the other half were different.

Each of the character judgment (i.e., orthographic, homophone, font size) task contained four 30-s experimental blocks and five 14-s rest blocks. In each trial, a pair of characters was exposed synchronously for 2000 ms, one above and one below a fixation crosshair (see Supplementary Fig. 2), followed by a 1000 ms blank time for subjects to give response. Subjects practiced for each task before scanning.

### fMRI data acquisition

All data were obtained on a SIEMENS PRISMA 3-Tesla scanner in the Imaging Center for Brain Research at Peking University. We collected fMRI data using an EPI sequence with the following parameters: axial slices = 33, thickness = 3.5 mm, matrix = 64 × 64, time of repetition = 2000 ms, time of echo = 30 ms, flip angle = 90°, field of view = 224 × 224 mm^2^. T1-weighted image was also acquired for each subject with 192 contiguous sagittal slices of 1-mm thickness and 7° flip angle. Time of repetition was 2530 ms, and time of echo was 2.98 ms. The acquisition matrix was 256 × 224 with voxel size of 0.5 × 0.5 × 1 mm^3^.

### fMRI data analysis

We used the Statistical Parametric Mapping software package (SPM12) (http://www.fil.ion.ucl.ac.uk/spm) for preprocessing. The functional images were first motion-corrected and then normalized to Montreal Neurological Institute (MNI) space by using T1 image-unified segmentation. Then functional volumes were resampled to isotropic 3 mm^3^ voxels, and spatially smoothed with a 6-mm full width at half maximum isotropic Gaussian kernel. In the first level analysis, the BOLD signal was modeled with the general linear model at each voxel, yielding contrast maps of activation for each subject. Group analysis including all contrast maps of subjects was performed using one-sample *t* tests to show regions of activation involved in each task for each group. Then the group contrast map was calculated using independent two-sample *t* test. We used a voxelwise and nonparametric permutation test (10,000 permutations) with TFCE correction (*p* < 0.05, cluster size >20), which was implemented in the TFCE Toolbox for SPM12 (http://www.neuro.uni-jena.de/tfce/). To filter out the regions with positive activation during the tasks, the result of two-sample *t* test in each task was masked with the union of one-sample *t* test contrast maps (voxel-level *ps* < 0.05) for two groups. The results were visualized on the smoothed template surface of International Consortium for Brain Mapping (ICBM) 152 using BrainNet Viewer^64^.

### Brain-behavior correlation analysis

We further examined the relationship among children’s reading performance and their brain activation patterns in the fMRI tasks. Beta values were extracted from spheres with 6-mm radius centered the peak coordinates for regions, which showed significant group effects in reading comprehension and orthographic judgment. In reading comprehension, we conducted correlation analyses between the beta value of the left middle frontal gyrus (BA 9/46; *x* = −48, *y* = 27, *z* = 30) and offline character recognition score, and between the beta value of the left inferior frontal gyrus (BA 44/45; *x* = −42, *y* = 15, *z* = 12) and offline character recognition score. In orthographic judgment, we conducted a correlation analysis between the beta value of the right fusiform gyrus (BA 37; *x* = 36, *y* = −39, *z* = −18) and orthographic awareness score.

### Functional connectivity

*Preprocessing*: The aim of our functional connectivity analysis was to examine the network of brain regions engaged in different elements of Chinese reading. Functional connectivity analyses were performed with DPABI^65^. Before functional connectivity analysis, several nuisance variables including six rigid-body motion parameters and their first-order temporal derivatives, the averaged signal from white matter and ventricles, and the global signal were removed by multiple linear regression analysis in DPABI.

*Definition of regions of interest*: Seven regions of interest were selected according to the peak coordinates reported in previous studies for each functional connectivity analysis (see Table 3)^36–38^, and spherical seeds were created with 6-mm radius in Montreal Neurological Institute coordinates, including the left middle frontal gyrus (*x* = −49, *y* = 20, *z* = 29), left inferior frontal gyrus (*x* = −53, *y* = 27, *z* = 16), left superior temporal gyrus (*x* = −53, *y* = −31, *z* = 9), left middle temporal gyrus (*x* = −59, *y* = −42, *z* = 3), left intraparietal sulcus (*x* = −30, *y* = −58, *z* = 48), left fusiform gyrus (*x* = −45, *y* = −62, *z* = −8), and right fusiform gyrus (*x* = 45, *y* = −62, *z* = −8).

*Functional connectivity analysis*: For each subject, the regional time course was calculated by averaging the time series of all voxels within the regions of interest. Then, the time course for each region of interest was correlated with every other region to generate the functional connectivity matrix (Fisher *r*-to-*z* transformed). For each task, one-sample *t* test was first conducted for each group. All significantly positive connections observed in at least one group of subjects in one-sample *t* test (*ps* < 0.05) were further compared between groups. Group contrast was calculated using independent two-sample *t* test with the FDR correction method and the *q*_corrected_ values after correction were presented.

### Voxel-based morphometry

*Preprocessing*: The above analyses revealed significant differences in brain activations and functional connectivity patterns between two groups of pinyin typists. To further investigate whether there were structural differences between the two groups of children in the brain regions responsible for Chinese reading, we performed a voxel-based morphometry (VBM) analysis to determine possible brain structure differences between two groups of children. Data were analyzed with FSL-VBM^66^ using FSL^67^. First, subjects’ T1-weighted images were brain-extracted using BET to remove non-brain tissues. Then, tissue type segmentation was carried out. The resulting gray matter partial volume images were then aligned to ICBM152 standard space using the affine registration. The resulting images were averaged to create a study-specific template, to which the native gray matter images were then non-linearly re-registered. The registered partial volume images were then modulated by dividing the Jacobian of the warp field. All modulated registered gray matter images were then concatenated and smoothed with an isotropic Gaussian kernel with a kernel of 4 mm.

*VBM analysis*: Since previous studies have indicated the importance of the left middle frontal gyrus in Chinese reading and reading acquisition, we examined the group difference in this brain region following a priori hypothesis as in previous research^30^. For statistical analysis, a small volume correction analysis procedure was applied to the left middle frontal gyrus (BA 9, *x* = −32, *y* = 31, *z* = 28, Talairach atlas; 20 mm region of interest), which was previously shown to be involved in reading in Chinese children^30^. The general linear model was used to compare voxelwise differences in gray matter volume between more frequent and less frequent pinyin users. Group differences in gray matter volume were calculated with permutation-based nonparametric testing (using a gap test) to find voxels that differed between the two groups. Nonparametric statistics were performed using “randomize” with 10,000 permutations and using the TFCE-correction at *p* < 0.05.

### Further analysis of pinyin typing effects

The effect of typing time might be contaminated by the effects of possible confounding variables, such as the total time spent on e-devices and character recognition scores of both groups, which showed group differences. Therefore, we conducted a regression analysis by adding sex, age, grade, nonverbal IQ, parental education (i.e., the average of mother and father education levels), head motion (i.e., the average of six rigid-body motion parameters), offline reading time, total time on devices, character recognition score, and pinyin typing group as covariates. The dependent variables included averaged voxel signals within regions which showed an effect of typing time in brain activations and VBM analyses, and functional connectivity strengths which showed an effect of typing time in functional connectivity analyses.

### Reporting summary

Further information on research design is available in the Nature Research Reporting Summary linked to this article.

## Supplementary information

supplementary information

reporting summary

## Data Availability

The data sets generated and/or analyzed during this study are available from the corresponding author on reasonable request.

## Code availability

The code used for data analysis is available from the corresponding author on reasonable request.
