# Supplementary material for: Children’s neurodevelopment of reading is affected by China’s language input system in the information era
Source: NPJ Sci Learn. 2020 Apr 3;5:3. doi: 10.1038/s41539-020-0062-0 (PMC7125128; doi:10.1038/s41539-020-0062-0)
Supplement: Supplementary file 1 — supplementary information [file 41539_2020_62_MOESM1_ESM.pdf]

Supplementary Information

Children's neurodevelopment of reading is affected by China's language input system in the information era

Wei Zhou, Veronica P. Y. Kwok, Mengmeng Su, Jin Luo*, and Li Hai Tan*

* Please send correspondence to: Jin Luo or Li Hai Tan.

Email: luoj@cnu.edu.cn; tanlh@sions.cn

Supplementary Method

Examples of the materials in reading comprehension, homophone judgment and orthographic judgment tasks were shown in Supplementary Figure 1 and Supplementary Figure 2.

Story	Translation
曾经有一个忠诚的有天赋的园丁，	There used to be a loyal and gifted gardener.
他一生都在照顾主人的花园。	He took care of the master's garden all his life.
主人却对他的优点和天赋视而不见。	The master turned a blind eye to his merits and gifts.
这个园丁的努力总是得不到认可。	The gardener's efforts were always not recognized.
直到有一天庄园经历了暴风雨。	Until one day the manor went through a storm,
园丁巧妙地对花园进行了重新修建。	the gardener cleverly rebuilt the garden.
主人终于认识到了园丁的忠实和聪明。	The master finally realized the loyalty and gift of the gardener.
这个故事告诉我们你也许会被忽视，	This story tells us that although you may be ignored,
但是只要你相信自己坚持自己。	as long as you believe in yourself and insist on being yourself,
终究有一天你的优点会被人们认可的。	your advantages will be recognized by people one day.

Supplementary Figure 1. An example of the story and its translation.

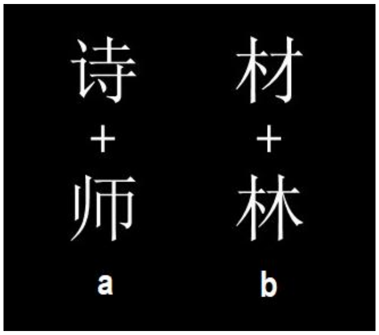

Supplementary Figure 2. Examples of experimental materials used in the homophone judgment and orthographic judgment tasks. a). Two homophone characters (pronounced shi1). b). Two orthographically similar characters.

Supplementary Results

Behavioral results. For offline behavioral test, we found that the scores of character recognition ($t(43) = -2.23$, $p = .031$), total time spent on e-devices ($t(43) = 4.96$, $p < .001$), and daily pinyin typing time ($t(43) = 6.36$, $p < .001$) were significantly different for less frequent pinyin typing users as compared to more frequent pinyin typing users. There was no significant group effect in orthographic awareness and phonological deletion (*ps* > .05). The correlation analysis indicated that the score of character recognition was positively correlated with the scores of orthographic awareness ($r(45) = .370$, $p = .013$) and writing time ($r(45) = .439$, $p = .003$).

Brain activation. One sample *t*-test (permutation with TFCE correction at $p < .05$) showed that reading comprehension in both groups elicited activation in the bilateral ventral visual cortex, left inferior frontal gyrus and left temporal cortex extending from posterior middle temporal gyrus to temporal pole. In addition, the activation in the less frequent pinyin typing group was observed in the left posterior superior temporal sulcus and the prefrontal cortex extending from the left middle frontal gyrus to left inferior

frontal gyrus (Supplementary Figure 3). Orthographic judgment in both groups elicited activation in the bilateral ventral visual cortex. The activation in the less frequent pinyin typing group was additionally observed in the left middle frontal gyrus and left superior parietal lobe (Supplementary Figure 4).

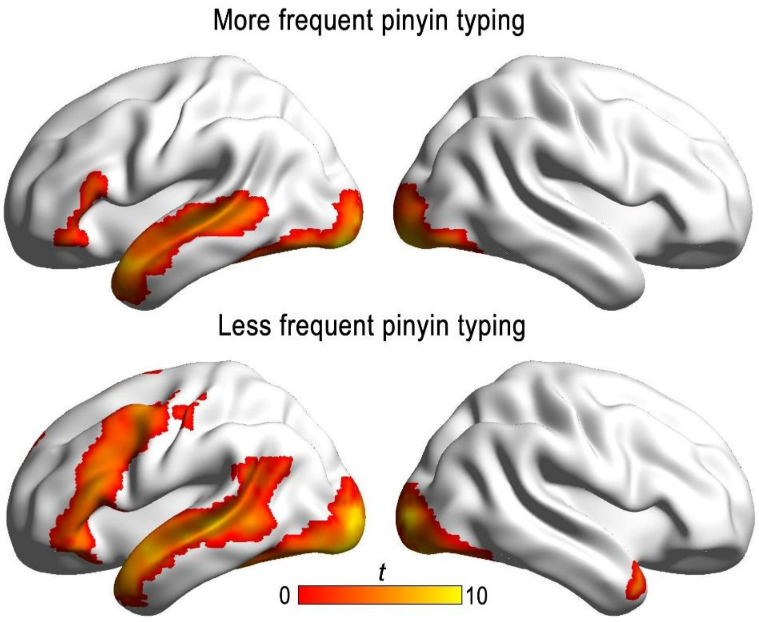

Supplementary Figure 3. The brain activation in reading comprehension for more frequent pinyin typing and less frequent pinyin typing users (permutation with TFCE correction at $p < .05$). The color bar denotes t-value.

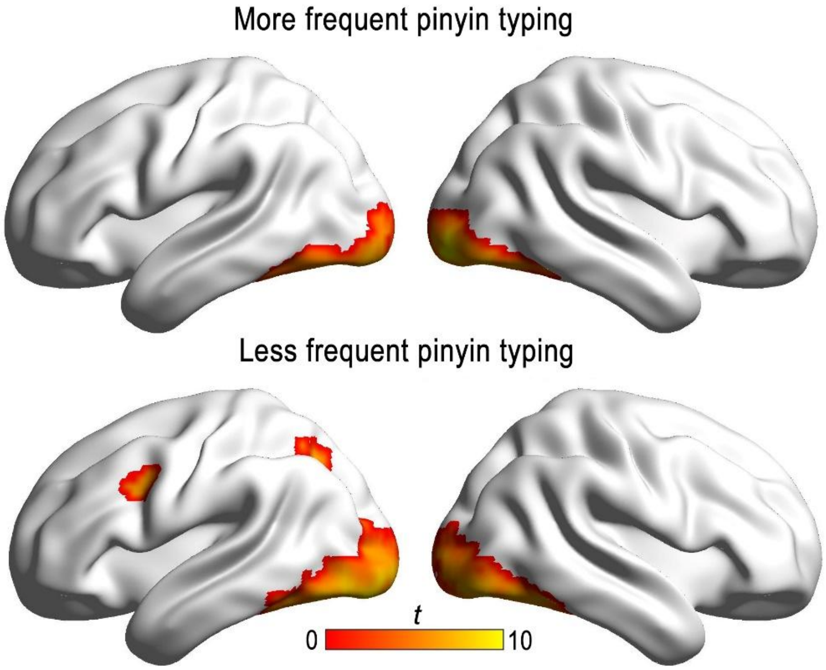

Supplementary Figure 4. The brain activation in orthographic judgment task for more frequent pinyin typing and less frequent pinyin typing users (permutation with TFCE correction at $p < .05$). The color bar denotes t-value.

Brain-behavior correlations. In the reading comprehension task, we found significantly positive correlations between offline character recognition and the brain activations of the left middle frontal gyrus ($r(42) = .477$, $p = .001$) and left inferior frontal gyrus ($r(42) = .405$, $p = .008$) in reading comprehension. There were positive correlations between offline character recognition and the activations of the left middle frontal gyrus and left inferior frontal gyrus within the more frequent pinyin typing group ($r(21) = .556$, $p = .009$; $r(21) = .380$, $p = .090$) but not within the less frequent pinyin typing group ($ps > .05$) (Supplementary Figure 5). We also found that there was a positive correlation between writing time and the brain activation of the left inferior frontal gyrus ($r(42) = .340$, $p = .034$). In the orthographic judgment task, there was a positive correlation

between orthographic judgment and the brain activation of the right fusiform gyrus ($r(41) = .367$, $p = .018$) (Supplementary Figure 6). The correlations between orthographic judgment and the brain activation of the right fusiform gyrus within less and more frequent pinyin typing groups were in a similar numerical trend but not significant ($ps > .05$).

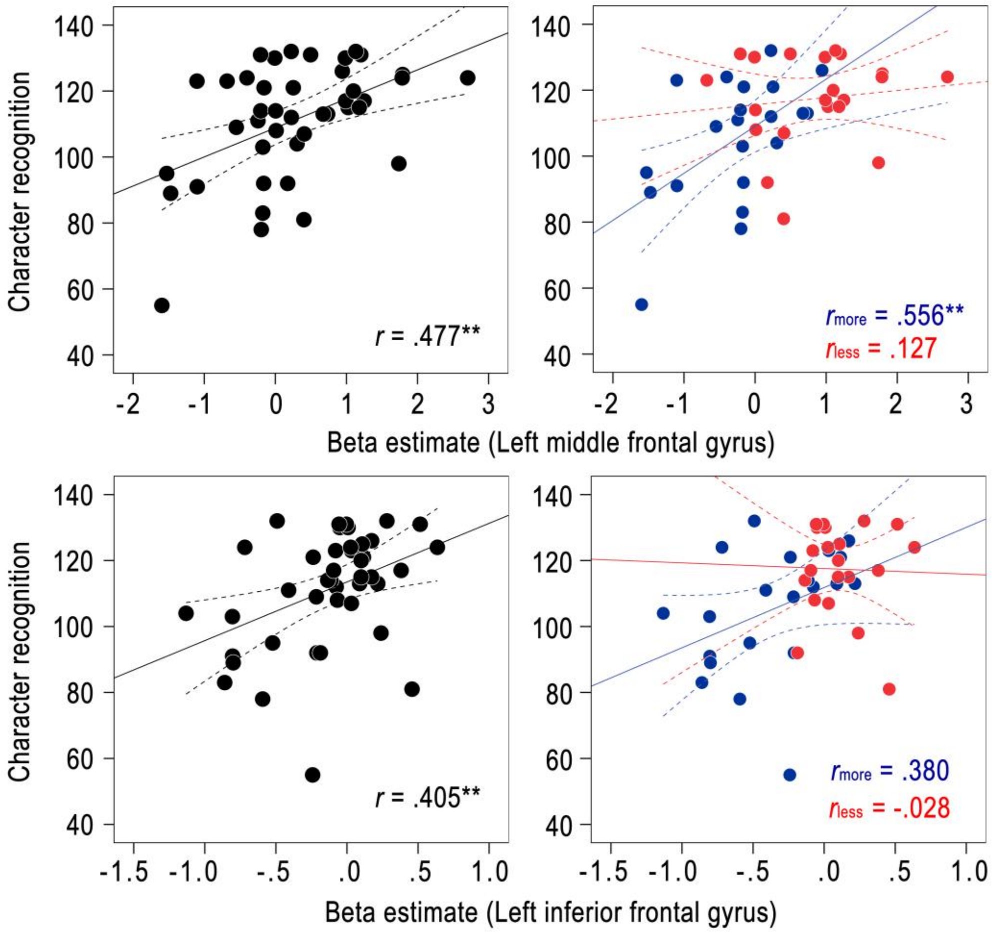

Supplementary Figure 5. The scatter plots of the correlations between character recognition and the activations of the left middle frontal gyrus and left inferior frontal gyrus in reading comprehension. The dotted lines indicate 95% confidence interval. ** $p < .01$.

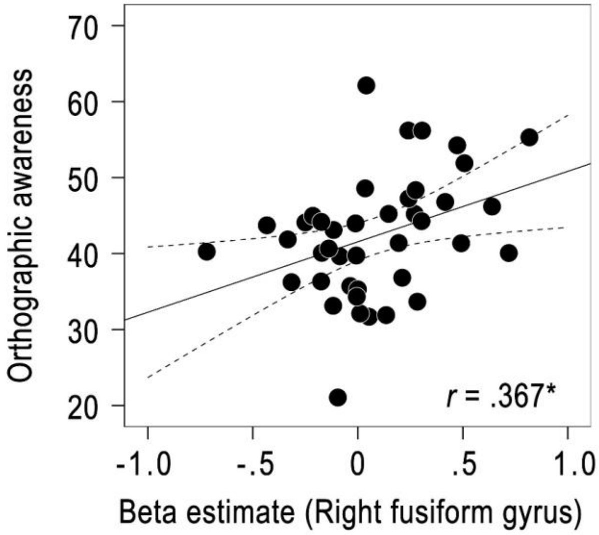

Supplementary Figure 6. The scatter plot of the correlation between orthographic awareness and the activation of the right fusiform gyrus in the orthographic judgment task. The dotted lines indicate 95% confidence interval. *$p < .05$.

Functional connectivity. We selected reading-related regions with the peak coordinates reported in previous studies[33-35] as regions of interest and computed functional connectivity with FDR correction among these regions. In the reading comprehension task (Supplementary Figure 7), we found that there were positively significant functional connections between the following regions: left middle frontal gyrus - left inferior frontal gyrus ($r_z(21) = .56$, $q_{corrected} < .001$; $r_z(21) = .37$, $q_{corrected} < .001$), left middle frontal gyrus - left intraparietal sulcus ($r_z(21) = .36$, $q_{corrected} < .001$; $r_z(21) = .18$, $q_{corrected} = .014$), left inferior frontal gyrus - left middle temporal gyrus ($r_z(21) = .42$, $q_{corrected} < .001$; $r_z(21) = .32$, $q_{corrected} < .001$), left middle temporal gyrus - left superior temporal gyrus ($r_z(21) = .26$, $q_{corrected} < .001$; $r_z(21) = .12$, $q_{corrected} = .050$), and left fusiform gyrus - right fusiform gyrus ($r_z(21) = .42$, $q_{corrected} < .001$; $r_z(21) = .46$,

$q_{corrected}$ < .001) for both less and more frequent pinyin users. Less frequent pinyin typing group exhibited an additional functional connection of left middle frontal gyrus - left middle temporal gyrus ($r_z(21) = .29$, $q_{corrected} < .001$). More frequent pinyin typing group showed additional functional connections of left intraparietal sulcus - left fusiform gyrus ($r_z(21) = .22$, $q_{corrected} < .001$), left middle temporal gyrus - right fusiform gyrus ($r_z(21) = .12$, $q_{corrected} = .039$), and left intraparietal sulcus - right fusiform gyrus ($r_z(21) = .10$, $q_{corrected} = .028$).

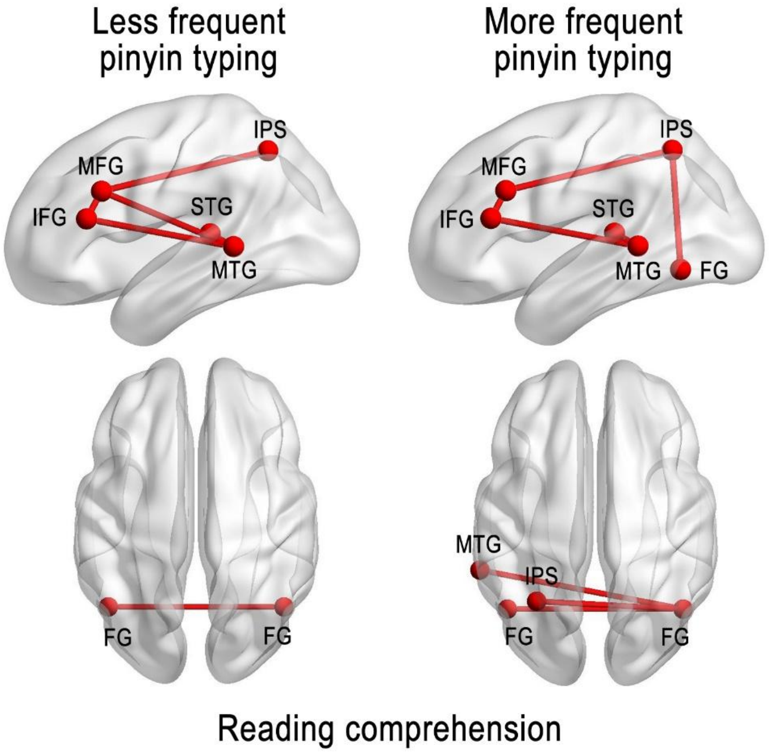

Supplementary Figure 7. Significant functional connectivity for less frequent and more frequent pinyin typing groups in the reading comprehension task after FDR correction ($qs_{corrected} < .05$). Note. FG = Fusiform gyrus. IFG = Inferior frontal gyrus. IPS = Intraparietal sulcus. MFG = Middle frontal gyrus. MTG = Middle temporal gyrus. STG = Superior temporal gyrus.
